# An Account of an Epidemic Fever That Prevailed in Cornwall in the Year 1788

**Published:** 1789

**Authors:** William May

**Affiliations:** Extra-Licentiate of the College of Physicians, London; and Physician at Truro in Cornwall


### LONDON MEDICAL JOURNAL.

### I. *An Account of an epidemic Fever that prevailed in Cornwall in the Year* 1788. *Communicated in a Letter to Dr.* Simmons, *F. R. S. by* William May, *M. D. Extra-Licentiate of the College of Physicians, London; and Physician at Truro in Cornwall.*

ABOUT the latter end of April, or the beginning of May, 1788, an epidemic made its appearance in this neighbourhood. The weather was rather warm than otherwise, and had been so, with little variation, for a few weeks preceding. At St. Ives, in the western part of this county, and in other small towns in various parts of it, a malignant fever had, for near two years past, been exceedingly rife among the poorer inhabitants, and carried off great numbers of them *. During this time also the small pox

* It deserves to be remarked, that in these places, owing to a failure, for the last four or five years, in the pilchard fishery, upon which the poorer inhabitants principally depend for their pox had been occaſionally epidemical in ſeveral parts of the county.

This epidemic was truly a fever of the typhus type, though accompanied in moſt inſtances, eſpecially at the attack, with ſymptoms of an inflammatory tendency. The tongue, during the firſt five or ſix days, was covered with a whitiſh mucus; the countenance was rather turgid, and the pulſe neither weak nor conſiderably accelerated. The thirſt was ſeldom conſiderable, the belly for the moſt part was coſtive, and the ſkin parched and dry.

In ſome caſes ſlight rigors were obſervable, followed, as in ordinary caſes of pyrexia, with an increaſe of heat, and ſometimes with ſweating; but this ſeldom happened: when, however, a determination to the ſkin took place, it was productive of great relief.

ſupport, a conſiderable increaſe of poverty had taken place amongſt them. The conſequences of this muſt have been a decreaſe of animal food, and the want of other things neceſſary for their ſuſtenance. To this circumſtance, as no ſpecific contagion could be traced, we may aſcribe the riſe of this formidable diſeaſe. I am indebted to my friend, the Reverend Mr. Townſend, of Pewſey in Wiltſhire, for ſome information reſpecting the ſame diſeaſe, evidently produced by the ſame remote cauſe, in his neighbourhood.

There

There was confiderable oppreffion at the breaft, with great and general languor; the head was loaded, and there was conftant pervigilium.

A difpofition to delirium fhewed itfelf early in the difeafe, fometimes fo foon as the third, but more commonly about the fourth or fifth day. The difeafe was marked with no other fymptoms of confequence till towards the fixth or feventh day, when the moft unequivocal appearances, of a typhus type, pretty uniformly fhewed themfelves. The countenance at this period became pale and dejected, the frequency and weaknefs of the pulfe greatly increafed, the colour of the tongue changed to a brownifh caft, marks of imbecillity were more evident, and, if a ftate of abfolute delirium did not exift at this time, there was always a ftate of pervigilium and inquietude prefent, which evinced confiderable difturbance of the nervous fyftem, and denoted fome derangement of the functions of the brain.

Among the anomalous affections, a dyfpnœa was frequently a moft troublefome fymptom, which, together with pain in fome part of the cheft, feemed to indicate thoracic inflammation; tion; and I was, in two or three inftances, nearly deceived by thefe appearances.

The difeafe paffed through whole families, and not unfrequently fpread throughout a neighbourhood, efpecially where it happened to affect a town or village fituated low, and confequently excluded from a free ventilation. It feemed to attack indifcriminately perfons of all ages and conftitutions, and was fcarcely, and I think not at all, lefs violent in robuft and ftrong than in weak and emaciated perfons; nor did it vary greatly in its duration, which was, in general, more diftinctly marked than in any cafes I had ever before met with. The changes alfo during the progrefs of the difeafe were marked with more uniformity than had before occurred to me. I attended to thefe circumftances with much precifion, having been accuftomed to great fcepticifm with refpect to the doctrine of critical days; and I could difcover that exacerbations conftantly happened towards the evening of the ninth, eleventh, and fourteenth days, which were followed by a manifeft apyrexia. In fome few inftances, indeed, fymptoms of convalefcence appeared on the latter of thefe days, but more commonly the feventeenth put an end to the diforder. Such was the uniformity nity with which this happened, that in many cafes, when the fourteenth day had paffed without any permanent remiffion of fever, I ventured to prognofticate, with much confidence, with refpect to approaching convalefcence, and was rarely deceived. Now and then the difeafe extended itfelf beyond the feventeenth day, efpecially where fome petechiæ had made their appearance; and where this happened I confefs that I could not, with any accuracy, afcertain any ftated time of its termination, which fometimes did not take place till towards the latter end of the fourth week.

The treatment that I, in general, adopted, was briefly as follows: — An emetic of ipecacuanha was firft exhibited, and the belly opened by fome gentle laxative, fuch as manna and cream of tartar, or a clyfter. The bark was then immediately adminiftered in fuch form as beft agreed with the ftomach of the patient. In general, the bark in fubftance, combined with fome aromatic, did very well, occafionally accompanied with fmall dofes of the fpiritus Mindereri or miftura camphorata. To obviate the determination to the head, blifters were applied to the back and arms, which were always found highly beneficial in abating the difturbance and erethiſm of the nervous ſyſtem; moderate doſes of laudanum, given towards the evening, contributed in no ſmall degree to this effect, and, even where there was a comatoſe tendency, were productive of material benefit. Wine was allowed in liberal quantities; and, with the repetition of clyſters, once in twelve hours, or thereabouts, to obviate coſtiveneſs, every part of the tonic plan was adopted, and ſeduloufly perſevered in. Among other things, the bodies of patients were occaſionally not only expoſed to a free air, but waſhed with cold water once in twelve hours, and particular attention was paid to repeated changes of their linen and bed clothes. The degree of ſucceſs which attended this method of treatment does not frequently occur; for of near fifty caſes that fell under my obſervation two only terminated fatally; and it is remarkable, that in one of theſe caſes a pain in the right hypochondrium had been miſtaken, before I ſaw the patient, for a ſymptom of hepatitis, and upon this idea bleeding and antimony had been preſcribed within twenty four hours of the patient's death. I have before obſerved that a pain in the ſide ſometimes happened, with a difficulty of breathing, and, though but very ſeldom, a trifling cough: all theſe

ſymptoms,

ſymptoms, however, conſtantly yielded to the tonic and antiſpaſmodic methods already deſcribed.

That this diſeaſe was truly a fever of the typhus type cannot, I think, be doubted, although, from the increaſed action which took place at its beginning, it put on an appearance of inflammatory diatheſis. This is, indeed, the true character of that fever which Dr. Cullen has deſcribed under the name of Synochus, and which he afterwards confeſſes to be no other, in his opinion, than a variety of typhus. This is his definition of the diſeaſe—"Morbus contagioſus. Febris ex ſynocha et typho compoſita: initio ſynocha; progreſſu, & verſus finem, typhus *."—I cannot, however, believe that the appearances of fulneſs, together with ſome hardneſs, in the pulſe in theſe caſes, are really ſymptoms of any purely inflammatory tenſion; for it has always been found that bleeding and the antiphlogiſtic regimen are peculiarly hurtful in affections of this kind; and it has frequently been obſerved by Dr. Willis, Dr. Huxham, and others, that even the ſmalleſt quantity of blood, drawn upon the ſecond and third

* Synopſis Noſ. Method.

days of the difeafe, has precipitated patients into a putrefcent condition, accompanied with a degree of debility out of which the moft powerful alexipharmacs were infufficient to roufe them *. I recollect having feen a lamentable inftance of this—A fever, which was clearly of this particular fpecies, became epidemical in a fmall neighbourhood; contrary to that whofe hiftory I have attempted above, its attack was confined principally to perfons advanced in life. The fymptoms of inflammation, as was fuppofed, were urgent at the beginning, and a dyfpnœa and turgid countenance fuggefting the probability of the exiftence of pneumonic congeftion and phlegmafia, the lancet was freely ufed. The

* Vide Huxham de Aëre & Morbis Epidemicis; & Willis de Febribus, (cap. xv.) where the following cafe is related:—"Noviffimo autumno, vir robuftus, habitûs corporis athletici, "vultûs tamen pallidi, & temperamenti frigidioris, in febrem "incidit: die fecundo cum calore & fiti, doloreque in lum-"bis immaniffimo, torquebatur: cum fanguinem *in exiguâ* "*quantitate* mitti prefcriberetur, pfeudo-chirurgus accerfitus "ipfum fere ad fefquilibram detraxit: paulo poft æger fudore "frigido perfundi, viribus fubito collapfis, rigore, pulfu de-"bili, inæquali, & crebra leiputhymia tentari cæpit," &c.—It may be objected that this is not a cafe in point, as the fever proved to be truly a variolous one. I have the authority, however, of Sydenham and Huxham for the applicatien of it.

confequences consequences were, as I have related, sudden prostration of strength to an alarming degree, and in most of the cases a premature appearance of putrescence. Whether from the original violence of the disorder, or from the want of its real nature being discovered, I will not pretend to determine, but the fact is too interesting to be withheld, namely, that of ten or eleven persons who were affected with the disease not one of them recovered *.

It is a matter of important inquiry how far the synochus, as Dr. Cullen has defined it, deserves, in a practical view, to be considered as different from the typhus. Dr. Cullen, with his usual candour, expresses his doubts about the propriety of making such a distinction, and they are well entitled to the attentive consideration of practitioners : — " Cum plures febres," says he, " nec inflammatoriæ, nec nervosæ " ex omni parte sint, neque idcirco vel ad

* This fact, melancholy as it is, does not stand alone. Speaking of a similar epidemic, under similar treatment I believe, Dr. Willis (de Febribus, cap. xiv.) says, " Memini in quibusdam villis, seniores fere omnes hoc anno vitâ " abreptos, ut vix superessent, qui traditionibus antiquitus " acceptis, Paroeciæ mores, & privilegia tueri potuerint."

" fynocham vel ad typhum facile referendæ; " genus fynochi, cujus typus hifce regionibus " frequens confpicitur, hic inferui. Inter ty- " phum tamen, & fynochum, limites accuratos " ponere non poffum, & an revera pro diverfis " generibus habenda, vel pofitis diverfis, utri " eorum fynonyma auctorum referenda, funt, " dubito *."—Again, in his Firft Lines, he expreffly declares his opinion, that there is no foundation for the diftinction, in thefe words: " I am difpofed to believe that the fynochus " arifes from the fame caufe as the typhus, and " is therefore only to be confidered as a variety " of it †." — With thefe fentiments, it may be expected, that, if we fhould be favoured with another edition of his valuable Synopfis, the fynochus will not be permitted to ftand as a peculiar genus of fever, but will be claffed among the varieties of typhus, of which it is unqueftionably a fpecies. Boerhaave, in his excellent Aphorifms, has given the defcription of a fever which very nearly refembles the epidemic above defcribed: — " Synochus putris dicta fuit, quæ " debetur caufis inflammatione fimplici majo-

* Synopf. Nof. Meth. Vol. II. page 79.

† Firft Lines of the Practice of Phyfic, Vol. I. page 126.

" ribus,

" ribus, viſcerum obſtructioni, cutis oppilatio-

" ni, & capillarium fere omnium, acrimoniæ

" vero acutiori, ſæpe prorſus ſingulari *."—Vogel has ſomewhat differently deſcribed it, and, except that the epithet *magnus* ſeems ill applied to the ſtate of the pulſe, has given an accurate definition of it: — " Synochus. Facie rubra, " cute humida, pulſu magno & frequenti, " criſi perfecta terminantur intra 21 dies †." — Sauvages alſo, following Galen's deſcription, has given a ſimilar account of the diſeaſe: — " Synochus. Decurſus ad duas treſve " ſeptimanas, cum pulſus robore ſanum, in " morbi ſtatu ſaltem, notabiliter ſuperante ‡."— It certainly does not happen in fevers incident to this climate, that the pulſe is found to exhibit marks of fulneſs and ſtrength after the ſixth or ſeventh day. In the hiſtory of the epidemic above related the pulſe uniformly became quick and weak before this period of the diſeaſe. Indeed the hard, ſtrong, and throbbing pulſe, that accompanied the ardent inflammatory fever, or

* Aphoriſmi de Cognoſcendis & curandis Morbis. 8vo. Leid. 1737.

† Vogelii Gen. Morborum, 16.

‡ Sauvages Gen. Morb. g. 81.

caufus, of the ancients, (which did not materially differ, in my opinion, from the fynochus of modern nofologifts) occurs but very feldom in European climates; and there is an obvious reafon why it fhould not. In every cafe of this fpecies of fever that I have obferved, the * condition of the pulfe has been exceedingly different from that which indicates a true inflammatory diathefis of the fyftem, and it was principally owing to my obfervation with refpect to this difference that I was cautious of adopting the antiphlogiftic regimen which, by the majority of fymptoms, appeared to be indicated. This particular ftate of the pulfe, in all inftances, and moft efpecially in cafes of the kind here related, fhould be feduloufly attended to, as being calculated to give a much better idea of the patient's real condition than all the other fymptoms taken in the aggregate; for, as a very fkilful Phyfician has faid, "Sine crebro, & diligente "pulfus examine, medicus nec prognofticon "rite faciet, nec pharmaceam tuto inftituet."

* The frequency of the pulfe, indeed, did not exceed, till towards the fifth day, ninety, or perhaps ninety-five ftrokes in a minute; and it might be faid, that, without being full or hard, or ftrong, it was only not a weak pulfe.

I am

I am persuaded that in every case of true synochus this attention to the state of the pulse would alone be sufficient to prevent the use of the lancet, which, even in the earliest stages of the disorder, never fails to aggravate its symptoms, and to protract to an enormous length, if not to render it absolutely incurable *. In the case of a young man, whose disease put on appearances, such as have been mentioned, of pneumonic inflammation, blood had been drawn before I saw him. I found him with every

* The following passage, relative to this subject, in Dr. Gregory's Conspectus Med. Theor. (Vol. II. p. 577) is so extremely judicious, and at the same time so applicable to what I am here advancing, that I must beg leave to transcribe it: — "Quin et in multis morbis ubi nimius sanguinis impe- "tus, et corporis calor a nimis vehemente cordis et arteriarum "actione fiunt, non semper convenit sanguinem mittere; ni- "mirum quia status iste saepe cum magna infirmitate conjun- "gitur, vel in hanc saltem brevi desinit, veluti in febribus "varii generis, cum intermittentibus, tum continuis: inter- "dum etiam idem status ad putredinem vergit, veluti in fe- "bribus quibusdam, aut variola confluente, aut angina ma- "ligna: in his igitur et similibus casibus, *utcunque urgentes* "*videantur*, sanguis vel cautissime detrahendus est, vel om- "nino non mittendus, quoniam talis exinanitio quae primis "morbi diebus, minus cauto medico prorsus necessaria vide- "retur, postea, per suos effectus infirmantes, non modo inu- "tilis, sed nocentissima plerumque foret."

 symptom

ſymptom of the prevailing epidemic, with a ſenſe of extreme oppreſſion about the præcordia, and in the loweſt ſtate of inanition and weakneſs. The blood bore not the leaſt marks of inflammation, but was of that kind which Huxham has deſcribed as indicating a looſened texture or broken craſis of the fluids. It was a mere gore, without the leaſt tendency to a ſeparation between the ſerous parts and craſſamentum, and had not the ſmalleſt portion of coagulable lymph on its ſurface. The diſeaſe, in this caſe, ran out to a great length, was accompanied with putrid appearances, and a degree of fatuity exiſted for ſome time after the febrile affection had totally diſappeared. In another caſe, which was under the care of a ſenſible Surgeon at Falmouth, a pain in the ſide, with oppreſſion and difficulty of breathing, were miſtaken for inflammatory ſymptoms, and bleeding and antimonials, with nitre, were directed by the Phyſician who was called in, imagining that the diſeaſe was a *paraphrenitis*. The conſequence was a ſpeedy deliquium, which was ſoon followed by convulſions, and their worſt effects. The practice which, in the beginning of this paper, I have briefly recommended, will, I am convinced, be found generally ſucceſsful cefsful in cafes of this kind, in which a fpeedy recourfe to the bark is of the utmoft confequence. It is not meant to be infinuated that it has any thing of novelty to recommend it; but it has a better recommendation: it has been adopted by the beft practitioners of this and other countries with fingularly good effects, and feveral phyficians of eminence have publicly recommended it*. One of thefe, Dr. Clark, of Newcaftle, has publifhed a number of cafes in which this medicine was exhibited, without regard to thofe nice cautions ufually obferved, with more than ordinary fuccefs: and we have lately been informed that Dr. James, who was a truly refpectable practitioner, adminiftered the

* I might add, in confirmation of the propriety of the practice I have here recommended, the teftimony of feveral refpectable practitioners. Mr. Grant, a Surgeon of extenfive practice at St. Auftle, has informed me that of upwards of a hundred cafes which he met with in the courfe of the laft fummer, and which were treated in the manner above mentioned, not more than four or five terminated fatally; and he farther obferved, that bleeding and antimonials, which, in a few inftances, were adopted, produced the moft obvious bad effects. Mr. Rice alfo, of Eaft Looe, and Mr. Trevoffo, of Falmouth, gentlemen of great profeffional fkill, with whom I had an opportunity of attending feveral patients, had repeated occafions to make the fame obfervation.

bark, after having premifed a dofe or two of his celebrated antimonial, in moft cafes, and in liberal quantities *. With what fuccefs he did this it is not neceffary for me to add. With refpect to the time and manner of exhibiting this remedy, there are, however, fome cautions very neceffary to be obferved; for as in intermittent fevers there would be impropriety, if not danger, in throwing in the bark during the exiftence of a paroxyfm, fo, in cafes of the continued type, it may be improper to interfere with the operation of the vis medicatrix naturæ juft at thofe periods of the difeafe when confiderable exacerbations are expected to take place. I confider what Hippocrates has faid of food † to be exceedingly applicable to the adminiftering of bark where exacerbations are about to take place. That thefe exacerbations do happen, and that with a great degree of regularity, I am thoroughly perfuaded; and in the inftances

* Monro on Medical and Pharmaceutical Chymiftry.

† "Εν δὲ τοῖσιν παροξυσμοῖσιν ὑποστέλλεσθαι χρὴ· τὸ προστιθέναι "γὰρ βλάβη. καὶ ὁκόσα κατὰ περιόδους παροξύνεται, ἐν τοῖσι παρο-"ξυσμοῖσιν ὑποστέλλεσθαι χρή." *i. e* "In exacerbationibus *ci*-"*bum* fubtrahere oportet; exhibere enim noxium eft. Et quæ-"cunque per circuitus exacerbantur, in exacerbationibus fub-"trahere oportet."—Aphor. fect. i. aph. xi.

here here related they were generally followed by more or lefs of a determination to the fkin, abatement of the perturbance of the intellectual function, and other marks of apyrexia. The interval between thefe exacerbations is the time in which the bark, aided by the methods already fpoken of, fhould be induftrioufly employed, and, for the reafons mentioned above, I would, upon the acceffion of an exacerbation, inftead of the bark, fubftitute a fmall dofe of fome antimonial, by which a fpeedier determination to the fkin might be promoted, and confequently the paroxyfm fooner terminated: upon this the bark and the other parts of the tonic plan fhould again be had recourfe to, and in this feries would I proceed to the end of the diforder *.

In

* It is a matter of great fatisfaction to me to find this fuggeftion, with refpect to the manner of ufing antimony and the bark together, fanctioned by the refpectable authority of Dr. Skeete, one of the Phyficians of Guy's Hofpital: — " In " the beginning of fevers, where flight inflammatory appea- " rances are fometimes prefent, or, at leaft, where weaknefs " is not very apparent, I fhould have no objection to the occa- " fional ufe of antimonials, at the fame time that I would " embrace every opportunity of adminiftering the bark freely; " for

In local congeftions, blifters will be preferable to any other application, affifted by the warm bath, which is often neceffary for obviating the fpafmodic affections that fo frequently occur in thefe cafes.

Dr. Sydenham gave the name of variolous fever to an epidemic very fimilar to that which I have here defcribed, and which happened in the years 1667, 1668, &c.* Dr. Huxham alfo, in his work De Aëre & Morbis Epidemicis, has given an interefting account of an epidemic which bears ftill a greater refemblance to that which is the fubject of this paper. It prevailed at Plymouth in the month of July, 1729. The fmall pox were at that time rife in the neighbourhood. The diforder affected the ftomach and loins, and was attended with an oppreffion of the breaft, fighing, and great faintnefs. Bleeding, he fays, unlefs at the beginning, (when probably it had better have been omitted) feldom did fervice. Vomits were

" for both antimony and bark appear to me to poffefs fuch " efficacy in the removal of fevers, as no theory of fever, " nor of the operation of the remedies themfelves, can ena- " ble us to explain." — Skeete on the Bark, page 198.

* Vide Opera Sydenhami de Epidemicis.

highly neceſſary, and, afterwards, frequent bliſters gradually applied; gentle cardiacs, cinnabar, opiates, ſack whey, and diluting ſubacid liquors, drank plentifully, proved very beneficial. As ſoon as the ſigns of coction appeared, namely, a ſediment in the urine and a remiſſion of fever, the bark admirably aſſiſted the cure. If a coma or delirium happened in the courſe of the diſeaſe, there was occaſion to ſet cupping glaſſes on the neck and ſhoulders, to bleed, and immediately apply bliſters, eſpecially behind each ear and to the head, and forthwith inject a laxative clyſter. This comatoſe and delirious tendency was remarkable in the ſynochus whoſe hiſtory I have given, and was conſtantly alleviated by camphorated medicines, combined with an opiate, and bliſters. It is, indeed, a matter of ſurpriſe to me, that Huxham did not prefer theſe methods, which he has highly extolled, in caſes of the ſame kind, to cupping glaſſes and laxative clyſters; for I imagine it muſt be pretty obvious that in a diſorder where "gentle cardiacs, opiates, and "ſack whey," were indicated, there could not have been occaſion for bleeding or alvine evacuations, methods that can only be neceſſary in plethoric and inflammatory diſeaſes. Indeed, in another another publication he has ſpoken of the ſame medicines, in the ſame caſes, in a ſtile of animated eulogium:—" Camphor, by its ano-" dyne, demulcent quality, is vaſtly ſervicea-" ble in quieting the erethiſm, and bringing " on compoſure of ſpirits and eaſy ſleep; and, " when joined with an opiate, is the moſt cer-" tain ſudorific in nature;" adding, with his accuſtomed energy, that " the elixir paregori-" cum, not only in this reſpect, but in many " others, is a moſt noble medicine."—From this we may infer, that the giving anodyne medicines, or, as the diſciples of modern ſchools would call them, powerful ſtimulants and antiſpaſmodics, ſuch as camphor, opium, and wine, is not, as it has been ſuppoſed, a practice perfectly new. Indeed we may go farther, and we ſhall find that what has been pronounced a dangerous innovation, is really a moſt judicious and confirmed good practice, and founded upon authorities which are almoſt as old as phyſic itſelf; but it is not to theſe authorities I would have recourſe in defence of a practice which has been adopted upon better grounds than imitation of the ancients, namely, upon recent diſcoveries with reſpect to the medicines, and an improved pathology with reſpect to the diſ-

eaſe case in question. The enthusiastic declaration, however, of Asclepiades, " Utilitatem vini æ-" quari vix Deorum potentiâ," is much to my purpose, and is well followed by the cooler testimony of Dr. Cullen. " What are the stimu-" lants that may be most properly employed, I " am uncertain, as the use of them, in this age, " has been rare; but I am disposed to believe " that, of all kinds, wine is the best *."

It is not my design in this place to enter fully into a discussion respecting the use of these medicines; but I am induced to dwell upon the circumstance by the recollection of the difficulties I met with in many cases, to convince practitioners of the indispensable necessity of administering them, even where a determination to the head (which is only another expression for extreme irritability) was the most urgent symptom. In such instances, from apprehension of phrenitic inflammation, it is too common a practice to give laxatives, under an idea of making a revulsion, and unloading the vessels of the head, by a large purgation; and this is frequently done in the advanced stages of typhous

* First Lines of the Practice of Physic, 4th Edit. Vol. I. page 192. Vide also Huxham on Fevers, page 122.

fever. But it is a miſtaken notion that an inflammation of the brain can happen under ſuch circumſtances. A *delirium mite*, or *typhomania*, may, it is true, be accompanied with ſome turgeſcence of the veſſels of the head; but this is merely a collateral ſymptom, and is ſcarcely of conſequence enough to require the topical evacuation of cupping. The proximate cauſe of this ſymptom is materially connected with the general ſyſtematic affection, and requires the ſame general method of treatment, namely, an anodyne antiſpaſmodic and tonic plan. It was this ſtate of morbid excitement and erethiſm for which Huxham, with great force, and equal judgment, recommended the camphorated and paregoric medicines. I ſhall add a quotation from Dr. Cullen's Firſt Lines, in confirmation of my opinions with reſpect to the utility of anodynes and antiſpaſmodics in this particular ſymptom: — "It may be ſuppoſed, and on good "grounds, that wine has an operation analogous "to that of opium and ſome other narcotic "medicines. It may, indeed, be ſaid that we "can diſtinctly mark its ſtimulant power only, "which renders its effects in the phrenitic de"lirium manifeſtly hurtful, and in the mild "delirium, depending on debility, as remaka-

"bly

"bly useful. But in all this, the analogy with "opium is still obvious *."

Before I dismiss the subject I shall beg leave to subjoin a few remarks respecting bleeding, as it has been recommended by some authors in the species of fever which I have here treated of; *i. e.* in its earliest stage. The decision with respect to the propriety of this practice rests upon this plain question:—Is the disease, at any period, accompanied with a true inflammatory diathesis; or are not the appearances of inflammatory action at the beginning the fallacious re-action of the system, in consequence of the exertions of the vis medicatrix naturæ to obviate the effects of the remote causes of the disease? This is reducing a complex affair to an intelligible and simple question, which may satisfactorily be determined; for it is a well-known fact, that even in the most excessive state of weakness which the animal œconomy can suffer, this re-action of the system will sometimes assume a speciousness of inflammatory tendency, as happens in the advanced stages of typhous and hectic fevers: and Dr. Cullen very ingeniously explains the manner in which this takes place, in the 46th sec-

* First Lines, Vol. I. page 194.

tion of his Firſt Lines. He has further declared, as we have already noticed, that the limits between the ſynochus and typhus are difficultly to be aſcertained, and is diſpoſed to believe that they originate in the ſame cauſe; conſequently that they are to be removed by the ſame means. We have ſeen that Dr. Huxham met with the ſame diſeaſe in which bleeding was ſeldom uſeful, but, on the contrary, gentle cardiacs and opiates were required. Dr. Willis and Dr. Sydenham give us the ſame teſtimony, which is confirmed by Dr. Lind and the beſt practitioners of the preſent day. As a farther proof, the inſtances here related are laid before the public, among which, although, in the early ſtage, all of them exhibited appearances that might be denominated inflammatory, not one of them was treated with evacuant means, nor, I am thoroughly perſuaded, could have admitted of the uſe of them without endangering the life of the patient. I do not mean to aſſert, that inflammatory fevers, and ſuch as require large and repeated venæſections, do not frequently happen; I acknowledge that they do, though with much leſs frequency, I believe, than is generally imagined; for, even in this part of the kingdom, where, from the purity and invigorating qualities of the air, a confiderable predifpofition to phlogiftic difeafes may be fuppofed to exift, a true fynocha is a very rare difeafe. Where it does happen, bleeding in large quantities, and repeated till the fymptoms of inflammation difappear, is the only method upon which reliance can be placed, Let it not, therefore, be imagined that I would wifh to prohibit entirely the ufe of the lancet; By no means: I only defire to prevent the abufe of it, impreffed with the importance of the following caution, which I beg leave to recommend to the attention of thofe bold practitioners whofe temerity, in this refpect, may require fome reftraint: — " Semper memoria tenendum, tan-
" tam fæpe quafi puncto temporis, ab intem-
" peftiva fanguinis detractione, corpori inferre
" perniciem, quantam neque optimi medici
" peritia, neque omnia medicinæ fubfidia, pof-
" tea compenfare potuerint. Sanguis enim fe-
" mel miffus nunquam in venas redit, virefque
" cum illo amiffæ in variis morbis haud facile
" reficiuntur *."

*Truro,*

January 26th, 1789.

* Vide Gregory's Confpectus Med. Theor. Vol. II. p. 575.

